# Medicaid Continuous Coverage Requirement and Postpartum Hospitalization

**DOI:** 10.1001/jamahealthforum.2025.6872

**Published:** 2026-02-27

**Authors:** Giacomo Meille, Maria W. Steenland, Erica L. Eliason

**Affiliations:** 1 American Board of Internal Medicine, Assessment and Research, Philadelphia, Pennsylvania; 2 Center for Financing Access and Cost Trends, Agency for Healthcare Research and Quality, Rockville, Maryland; 3 University of Maryland, School of Public Health, College Park; 4 Center for State Health Policy, Rutgers University, New Brunswick, New Jersey

## Abstract

**Importance:**

In 2020, all US states adopted the Families First Coronavirus Response Act Medicaid continuous coverage requirement (CCR), which prevented disenrollment from Medicaid. This policy effectively extended pregnancy-related Medicaid eligibility beyond the previous end date of 60 days post partum.

**Objective:**

To determine whether the CCR was associated with a change in postpartum hospitalization rates for mothers covered by Medicaid during their delivery.

**Design, Setting, and Participants:**

This cohort study was conducted from December 2023 to March 2025 at all nonfederal acute care hospitals in 20 states using data from the Healthcare Cost and Utilization Project’s State Inpatient Databases from 2018 through 2021. Participants included mothers aged 18 to 55 years who had Medicaid-paid deliveries (based on the primary expected payer) from January 2018 to June 2021; they were followed up for 180 days post partum.

**Exposures:**

State-level postpartum uninsured rate among mothers with Medicaid-paid deliveries that occurred from 2018 to 2019 (before the CCR was implemented in 2020). Residency in states with an uninsured rate above the median was considered to be high exposure (ie, a greater share of the state population had the potential to benefit from the CCR) and residency in states with a preperiod uninsured rate below the median was considered low exposure.

**Main Outcomes and Measures:**

Probability of a mother being hospitalized 1 to 60 days post partum (covered under previous Medicaid eligibility rules) and 61 to 180 days post partum (newly covered under the CCR).

**Results:**

A total of 2 024 214 mothers (mean age, 27.5 [95% CI, 27.5-27.5] years) with Medicaid-paid deliveries were identified. The sample included 550 881 deliveries by Hispanic mothers (27.2%), 490 586 deliveries by non-Hispanic Black mothers (24.2%), 744 945 deliveries by non-Hispanic White mothers (36.8%), 200 639 deliveries by mothers of other races and ethnicities (9.9%), and 37 163 deliveries by mothers with missing race and ethnicity data (1.8%). Among the full sample, 931 452 mothers (mean age, 27.1 [95% CI, 27.71-27.2] years) resided in high-exposure states and 1 092 762 mothers (mean age, 27.8 [95% CI, 27.8-27.8] years) resided in low-exposure states. In adjusted difference-in-differences models, the rate of hospitalization per 1000 Medicaid-paid deliveries at 61 to 180 days post partum decreased (adjusted β coefficient, −1.4; 95% CI, −2.5 to −0.3) in high-exposure states compared with low-exposure states, a 10.9% decrease relative to the preperiod mean (12.9; 95% CI, 12.6 to 13.2) in high-exposure states. The analogous estimate for the hospitalization rates 1 to 60 days post partum was not statistically significant (−0.7 [95% CI, −2.0 to 0.5] per 1000 Medicaid-paid deliveries).

**Conclusions and Relevance:**

Findings of this study suggest that residency in high-exposure states was associated with a reduction in hospitalizations for mothers 61 to 180 days post partum (the period newly covered by extended Medicaid coverage). Extended Medicaid coverage may help to improve postpartum health and reduce adverse events among mothers with low income.

## Key Points

**Question:**

Was the Medicaid continuous coverage requirement (CCR) associated with changes in the postpartum hospitalization rate among mothers with Medicaid-paid deliveries (based on primary expected payer)?

**Findings:**

This cohort study identified 2 024 214 mothers with Medicaid-paid deliveries and found that, after the CCR was implemented, states that had higher rates of postpartum uninsurance before the CCR was implemented had larger declines in hospitalization rates 61 to 180 days post partum.

**Meaning:**

Findings of this study suggest that extended Medicaid coverage under the CCR was associated with a reduction in the postpartum hospitalization rate for mothers with Medicaid-paid deliveries in states that had higher rates of postpartum uninsurance before the CCR was implemented.

## Teaser

This cohort study assesses the association of the Medicaid continuous coverage requirement with changes in the postpartum hospitalization rate among mothers with Medicaid-paid deliveries.

## Introduction

After childbirth, preventive care and management of conditions are essential for improving health and decreasing the risk of adverse events, including preventable emergency department visits and hospitalizations.^1,2,3^ Postpartum health insurance is crucial for ensuring access to such care.^4^ Previous research found that the Patient Protection and Affordable Care Act (ACA) Medicaid expansions, which increased postpartum Medicaid coverage and use of postpartum outpatient care,^5,6^ were associated with reductions in postpartum hospitalizations.^7^

During the COVID-19 public health emergency (PHE) all US states adopted the Medicaid continuous coverage requirement (CCR), a provision of the March 2020 Families First Coronavirus Response Act. This provision required continuous enrollment of Medicaid recipients throughout the PHE, effectively extending pregnancy-related Medicaid eligibility nationally. Prior to the CCR, pregnancy-related Medicaid coverage ended 60 days post partum^8^; to retain Medicaid coverage thereafter, postpartum mothers needed to qualify under another pathway, such as parental coverage, which had lower income thresholds than pregnancy-related coverage in every state.^9^ Previous studies have found that this insurance expansion increased Medicaid retention and reduced uninsurance in the postpartum period.^10,11,12,13,14^

To our knowledge, studies have not yet examined whether the CCR was associated with changes in postpartum hospitalization rates.^10,11,12,13,14^ Increased insurance coverage may have improved access to primary care, thereby reducing preventable postpartum hospitalizations. However, increased insurance coverage also reduced out-of-pocket costs for hospitalizations, which may have increased risk-appropriate and discretionary postpartum hospitalizations. The net association of the CCR with postpartum hospitalizations is theoretically ambiguous and studying this relationship may provide insight into the policy regime that superseded the CCR. Although the CCR ended in 2023, as of January 2025, 48 states and Washington, DC, extended pregnancy-related Medicaid coverage to 12 months post partum under a provision of the American Rescue Plan Act of 2021.^15^

In this cohort study, we used a difference-in-differences (DID) design that used state variation in postpartum uninsured rates before the PHE to estimate the incremental association of the CCR with postpartum hospitalizations in high-exposure vs low-exposure states. We examined overall changes in hospitalization rates, differences by diagnostic category and expected payer of the postpartum hospitalization, and differences by patient race and ethnicity and county urbanicity.

## Methods

This cohort study was reviewed by a human protection administrator at the Agency for Healthcare Research and Quality, who determined that it did not constitute human participants research and did not require additional review by an institutional review board or informed consent. We followed the Strengthening the Reporting of Observational Studies in Epidemiology (STROBE) reporting guideline for cohort studies.

### Data Source

We used 2018 to 2021 data from the Agency for Healthcare Research and Quality’s Healthcare Cost and Utilization Project State Inpatient Databases. These data include all inpatient discharge records from nonfederal acute-care hospitals in participating states. Data include diagnoses, patient demographics, and primary expected payer (hereafter payer) for each discharge based on billing records. The actual payer was not observed and may differ.^16^ Previous studies have used birth certificate and hospital discharge data to evaluate the accuracy of hospital-reported expected payers. These studies were able to link 79% to 97% of records that were reported as paid by Medicaid by the hospital to a Medicaid enrollment record.^17,18,19,20^

### Study Population

We included encounters from 20 of 21 states that had encrypted patient identifiers that allowed each patient’s hospitalizations to be linked across the study years (eTable 1 in Supplement 1).^21^ We excluded Utah because Medicaid was expanded there at nearly the same time that the CCR was implemented. We identified hospitalizations for delivery among females aged 18 to 55 years based on diagnosis codes, procedure codes, and diagnosis-related group codes.^22^ We limited the study population to mothers with Medicaid-paid deliveries that occurred from January 2018 through June 2021. Each mother was followed up for 180 days after each delivery, allowing analysis of hospitalizations that occurred more than 60 days post partum while also including mothers who delivered during the first half of 2021. Every delivery during the sample period was included, which resulted in mothers with multiple deliveries being included multiple times.

### Outcomes

Our primary outcome of interest was the probability of a hospitalization 61 to 180 days after the delivery admission date (reported as the rate per 1000 Medicaid-paid deliveries). Under the CCR, Medicaid beneficiaries retained coverage during the 180-day follow-up period, but prior to the CCR, pregnancy-related Medicaid coverage ended at 60 days post partum. In exploratory analyses, we examined the probability of hospitalization 61 to 180 days post partum paid by private insurance, Medicaid, and self-pay (all within the sample of Medicaid-paid deliveries). Similarly, we examined the probability of hospitalization 61 to 180 days post partum with the most common principal diagnosis codes categorized based on the *International Statistical Classification of Diseases, Tenth Revision, Clinical Modification* codebook, with O representing pregnancy, childbirth, and the puerperium; K representing digestive diseases; F representing mental and behavioral disorders; and A-B representing infections and parasitic diseases (eTables 4, 5, and 6 in Supplement 1). Lastly, we stratified the analyses by patient race and ethnicity as recorded on the discharge record by the hospital, which is typically based on self-report but may be collected based on observation (Hispanic; non-Hispanic Black; non-Hispanic White; and other race or ethnicity, which included American Indian or Alaska Native, Asian, and other races or ethnicities) and by metropolitan vs nonmetropolitan patient county. Race and ethnicity were analyzed because rates of maternal morbidity and postpartum hospitalization vary by race and ethnicity.

### Study Design 

We adopted a DID design based on the degree to which a state’s population had the potential to benefit from the CCR. Mothers were affected by the CCR if they would have otherwise lost Medicaid coverage at 60 days post partum. Thus, our exposure variable was a state-level measure of the 2018 to 2019 postpartum uninsured rate for mothers with Medicaid-paid deliveries.

We constructed the exposure variable using 2018 to 2019 Pregnancy Risk Assessment Monitoring System (PRAMS) data for 14 states, which asks respondents about insurance at the time of delivery and 2 to 6 months post partum.^23^ These data were available for 14 states: Alaska, Arkansas, Iowa, Louisiana, Maryland, Missouri, Mississippi, New York, Oregon, South Dakota, Virginia, Vermont, Wisconsin, and Wyoming. For 6 states (California, Florida, Indiana, Nevada, South Carolina, and Tennessee) without the necessary PRAMS data (eTable 1 in Supplement 1), we imputed the exposure variable based on a closely related measure of postpartum uninsurance from the American Community Survey (ACS). The ACS measure was the 2018 to 2019 uninsured rate among individuals who had a birth in the past 12 months and reported an income less than their state’s Medicaid eligibility levels for pregnancy. The ACS measure of postpartum uninsurance correlated with the PRAMS measure (eFigure 1 and eTable 2 in Supplement 1). The postpartum uninsured rate from 2018 to 2019 was a reliable indicator of the change in postpartum uninsured rate after the CCR was implemented (eFigure 2 and eTable 3 in Supplement 1). We assigned the exposure variable based on the patient’s state of residence at the time of delivery, which could differ from the state the hospital was located in. We excluded patients who resided outside of the 20 study states at the time of delivery.

We grouped states based on the population-weighted median of the exposure variable, resulting in 12 states above the median (Arkansas, Florida, Indiana, Maryland, Missouri, Mississippi, Nevada, South Carolina, South Dakota, Virginia, Wisconsin, Wyoming) and 8 states at or below the median (Alaska, California, Iowa, Louisiana, New York, Oregon, Tennessee, Vermont). Our main DID model compared states that were above the median postpartum uninsured rate during the pre-PHE period (ie, high-exposure states in which a greater share of the population had the potential to benefit from the CCR) to states that were below the median rate (ie, low-exposure states). Recent literature examining this framework has shown that, under standard assumptions, including parallel trends at each level of exposure and no anticipatory effects, the traditional DID estimator recovers the difference in the association of the CCR with postpartum hospitalizations in the high-exposure vs low-exposure states.^24,25^ The preperiod included deliveries from 2018 to 2019, and the postperiod included deliveries from 2020 to 2021. Pregnancy-related Medicaid coverage for January 2020 deliveries would have ended in March 2020 under the 60-day regime. Thus, the January 2020 birthing cohort was the first to be affected by the CCR, which was implemented in March 2020.

### Statistical Analysis

We estimated linear probability models where the primary variable of interest was an interaction term between the high-exposure group and the postperiod (eMethods in Supplement 1). In all models, we included state and year fixed effects and controlled for Virginia’s 2019 ACA Medicaid expansion (no other included state expanded Medicaid in the sample period). In adjusted models, we also controlled for patient characteristics, delivery characteristics, and the COVID-19 case load. Controls included length of stay (1, 2, 3, ≥4 days), age at delivery (18-24, 25-29, 30-34, 35-39, and ≥40 years), race and ethnicity (Hispanic, non-Hispanic Asian, non-Hispanic Black, non-Hispanic White, other, missing), gestational age (<32, 32-36, 37-38, 39-40, and ≥41 weeks), cesarean delivery, COVID-19 diagnosis at delivery, the Hospital Service Area-level COVID-19 admission rate in the follow-up period, the Elixhauser Comorbidity Index Refined for readmission,^26^ an indicator for severe maternal morbidity that excluded transfusion,^22^ and the 2023 Rural-Urban Continuum Code (1, 2, 3, ≥4).^27^ We estimated SEs using wild bootstrapping, a more conservative approach than clustering by state.^28^ Indicator variables were used for missing race and ethnicity data (1.8% of deliveries) and for missing comorbidity index data in Wyoming hospitals, which did not report whether conditions were present on admission (0.31% of deliveries). Deliveries with any other missing covariates (0.1%) were excluded.

To assess the plausibility of our study design, we examined whether state hospitalization rates followed parallel trends in the preperiod by graphing trends in postpartum hospitalizations for the high-exposure and low-exposure groups and estimating event study models. We also estimated a continuous DID specification, which interacted each state’s preperiod postpartum uninsured rate with the postperiod.^29,30,31^ This model required additional assumptions, including a linear association between the preperiod postpartum uninsured rate and the change in hospitalizations.^32,33,34,35^ We graphed a scatterplot of this association at the state level (eFigure 5 and eFigure 6 in Supplement 1).

The implementation of the CCR coincided with the start of the COVID-19 pandemic, which also affected hospitalizations.^36,37^ In addition to controlling for the COVID-19 hospitalization rate, we performed a sensitivity analysis that excluded the initial months of the pandemic from our estimation sample because hospital use dropped substantially during this time. We also conducted sensitivity analyses that used only ACS data on preperiod uninsured rates. These data were available for all states but were a less direct measure of exposure to the CCR than the PRAMS data used in our main analysis. Finally, we conducted 2 placebo analyses. We estimated the association of the CCR with hospitalization rates within 1 to 60 days post partum, a period that was already covered by pregnancy-related Medicaid prior to the CCR. In addition, we estimated the association of the CCR with hospitalization rates for patients with privately insured deliveries, who should not have been affected by the CCR. Data were analyzed from December 2023 to March 2025 with Stata version18 (StataCorp). Statistical significance was set at α = .05. Differences in means for all characteristics were statistically significant at the 1% level due to large sample size.

## Results

We identified 2 024 214 mothers (mean age, 27.5 [95% CI, 27.5-27.5 years) with Medicaid-paid deliveries from January 2018 to June 2021 in the 20 study states. The sample included 550 881 deliveries by Hispanic mothers (27.2%), 490 586 deliveries by non-Hispanic Black mothers (24.2%), 744 945 deliveries by non-Hispanic White mothers (36.8%), 200 639 deliveries by mothers of other races and ethnicities (9.9%), and 37 163 deliveries by mothers with missing race and ethnicity data (1.8%). Mothers with multiple deliveries accounted for 20.5% of observations.

In the preperiod, patients residing in states with postpartum uninsured rates below the median had lower hospitalization rates within 60 days post partum (19.7 per 1000 Medicaid-paid deliveries) and 61 to 180 days post partum (9.9 per 1000 Medicaid-paid deliveries) compared with patients in states with postpartum uninsured rates above the median (21.8 per 1000 Medicaid-paid deliveries at 60 days post partum and 12.9 per 1000 Medicaid-paid deliveries at 61 to 180 days post partum) (Table 1). Patients residing in states with lower baseline postpartum uninsured rates vs those in states with rates above the median were more likely to be Hispanic (35.8% vs 16.6%) or non-Hispanic Asian (5.9% vs 2.1%) and were less likely to be non-Hispanic Black (17.4% vs 32.3%) or non-Hispanic White (32.0% vs 42.6%). Patients were similar in terms of age at delivery, gestational age, cesarean delivery rate, comorbidities, severe maternal morbidity rate, and length of stay. Patients in states with postpartum uninsured rates below the median were more likely to reside in a metropolitan county (90.6% vs 83.0%) and in a state that expanded Medicaid under the ACA (90.3% vs 32.9%) compared with patients in states with postpartum uninsured rates above the median.

**Table 1.** Characteristics for Mothers With Medicaid-Paid Deliveries From 2018 Through 2019 Stratified by State Postpartum Uninsured Rate

Characteristic	Mean (95% CI)	
	States with an uninsured rate at or below the median in 2018 through 2019^a^	States with an uninsured rate above the median in 2018 through 2019^a^
Total No. of mothers	643 878	548 598
Postpartum hospitalizations per 1000 Medicaid-paid deliveries		
Within 60 d	19.7 (19.3-20.0)	21.8 (21.4-22.2)
Within 61-180 d	9.9 (9.7-10.2)	12.9 (12.6-13.2)
Patient demographics		
Age, y	27.7 (27.7-27.7)	27.0 (27.0-27.1)
Hispanic, %	35.8 (35.7-36.0)	16.6 (16.5-16.7)
non-Hispanic Asian, %	5.9 (5.9-6.0)	2.1 (2.1-2.2)
non-Hispanic Black, %	17.4 (17.3-17.5)	32.3 (32.2-32.4)
non-Hispanic White, %	32.0 (31.9-32.1)	42.6 (42.5-42.7)
Other race or ethnicity, %^b^	7.1 (7.0-7.1)	4.5 (4.4-4.5)
Missing race or ethnicity, %	1.7 (1.7-1.7)	1.9 (1.8-1.9)
Clinical characteristics		
Gestational age, wk	38.3 (38.3-38.3)	38.1 (38.1-38.1)
Cesarean delivery, %	31.0 (30.9-31.1)	33.3 (33.2-33.5)
Comorbidity index	1.1 (1.1-1.1)	1.3 (1.3-1.3)
Severe maternal morbidity per 1000 Medicaid-paid deliveries	9.0 (8.8-9.3)	8.3 (8.1-8.6)
Length of stay, d	2.7 (2.7-2.7)	2.6 (2.6-2.6)
Patient location		
Metropolitan county, %	90.6 (90.5-90.7)	83.0 (82.9-83.1)
Medicaid expansion state, %	90.3 (90.2-90.4)	32.9 (32.8-33.0)

^a^ The study period 2018 through 2019 corresponds to the period before the Medicaid continuous coverage requirement was implemented. Difference between states above and below the median uninsured rate was statistically significant at the 1% level for all characteristics.

^b^ Other race or ethnicity includes American Indian or Alaska Native and other races or ethnicities except Black, Hispanic, and White.

The Figure shows the unadjusted trend in the hospitalization rate at 61 to 180 days post partum among patients with Medicaid-paid deliveries by delivery month stratified by high exposure and low exposure. During the postperiod (ie, deliveries from 2020 to 2021), postpartum hospitalization rates remained relatively stable in low-exposure states. In contrast, high-exposure states experienced a decline in postpartum hospitalization rates, which narrowed the difference in the postpartum hospitalization rate relative to low-exposure states. eFigure 4 in Supplement 1 shows a relative decrease in hospitalization rate during the postperiod among high-exposure states. eFigures 5 and 6 in Supplement 1 show the change in hospitalization rate vs the pre-PHE postpartum uninsured rate in high-exposure states, with an approximately linear association between the 2 variables.

**Figure.**
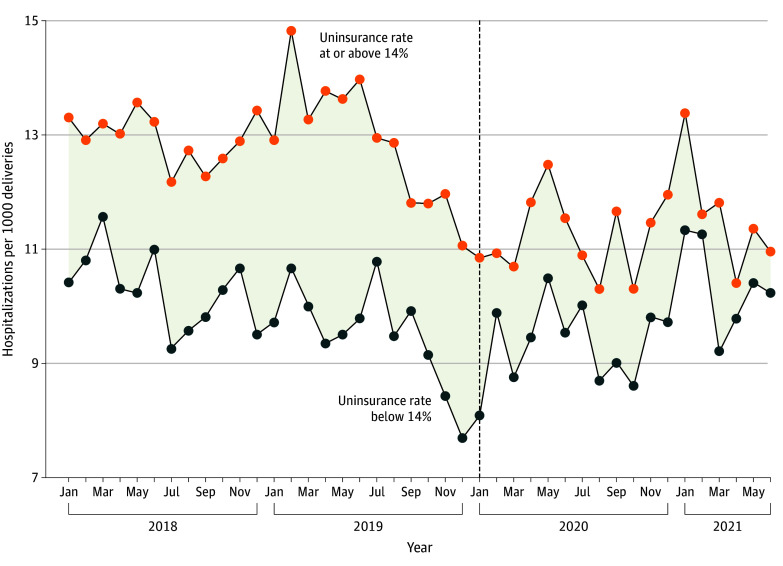
Line Graph of Hospitalizations 61 to 180 Days Post Partum Among Patients With Medicaid-Paid Deliveries Stratified by Preperiod State Uninsured Rate Dashed vertical line represents the beginning of the postperiod in January 2020. Patients delivering during the postperiod were affected by the Medicaid continuous coverage requirement. States with a 0% to 14% uninsured rate included Alaska, California, Iowa, Louisiana, New York, Oregon, Tennessee, and Vermont. States with a 14% or greater uninsured rate included Arkansas, Florida, Indiana, Maryland, Missouri, Mississippi, Nevada, South Carolina, South Dakota, Virginia, Wisconsin, and Wyoming.

In DID models, the rate of hospitalization at 61 to 180 days post partum per 1000 Medicaid-paid deliveries decreased more in states with preperiod postpartum uninsured rates above the median for patients with Medicaid-paid deliveries relative to states that had preperiod uninsured rates below the median (adjusted β coefficient, −1.4; 95% CI, −2.5 to −0.3) (Table 2). This DID estimate corresponded to a 10.9% decrease relative to the preperiod mean of the high-exposure states (12.9 per 1000 Medicaid-paid deliveries).

**Table 2.** Difference-in-Differences Estimates for the Association Between the Medicaid Continuous Coverage Requirement and Postpartum Hospitalization Rate per 1000 Deliveries^a^

Outcome	Estimate (95% CI)			
	States with an uninsured rate at or below the median, preperiod mean^b^	States with an above median uninsured rate, preperiod mean^b^	Unadjusted β coefficient^c^	Adjusted β coefficient^c^^,^^d^
Hospitalizations 1-60 d post partum	19.7 (19.4 to 20.0)	21.8 (21.4 to 22.2)	−0.4 (−1.5 to 0.7)	−0.7 (−2.0 to 0.5)
Hospitalization 61-180 d post partum	9.9 (9.7 to 10.2)	12.9 (12.6 to 13.2)	−1.2 (−2.1 to −0.3)	−1.4 (−2.5 to −0.3)

^a^ Includes 2 024 214 mothers who had deliveries with an expected payer of Medicaid.

^b^ Median uninsured rate of 13.9%.

^c^ Coefficients were estimated from a difference-in-differences model comparing changes in high-exposure vs low-exposure states. High exposure was defined as a preperiod postpartum uninsured rate above the median (13.9%).

^d^ Adjusted models controlled for delivery characteristics, patient demographics and comorbidities, and COVID-19 admission rates for the relevant follow-up period.

We obtained similar estimates using the continuous DID model for all included states (eMethods and eTable 7 in Supplement 1). Multiplying the model β coefficient (−9.7 [95% CI, −17.0 to −2.1] hospitalizations per 1000 Medicaid-paid deliveries) by the mean state-level uninsured rate in the preperiod (16.3%) yielded a mean decline of 1.6 (95% CI, −2.8 to −0.3) hospitalizations per 1000 Medicaid-paid deliveries, a 14.2% decline relative to the preperiod mean of 11.3 (95% CI, 11.1-11.5) per 1000 Medicaid-paid deliveries. Estimates were also similar when we excluded deliveries for which the follow-up period was during the initial months of the COVID-19 pandemic and when using estimates of uninsurance from the ACS (eMethods and eTables 8-10 in Supplement 1).

In our placebo analyses, we examined hospitalizations occurring 1 to 60 days after delivery, and found no evidence of changes during the postperiod in the raw trends in hospitalization rates (eFigure 7 in Supplement 1), the event study (eFigure 3 in Supplement 1), and the DID specifications (Table 2; eTable 7 in Supplement 1). We also examined patients with privately insured deliveries, who had substantially lower postpartum readmission rates compared with patients with Medicaid-paid deliveries (5.7 [95% CI, 5.5 to 5.8] vs 12.9 [95% CI, 12.6 to 13.2] hospitalizations 61 to 180 days post partum per 1000 deliveries in high-exposure states). Similar to the other placebo analyses, we found no evidence of changes in hospitalization rates for patients with privately insured deliveries (eMethods, eFigure 8, and eTable 11 in Supplement 1).

In exploratory analyses, we examined the payer for hospitalizations 61 to 180 days post partum among patients with Medicaid-paid deliveries (Table 3). The rate of self-pay hospitalization per 1000 Medicaid-paid deliveries declined in high-exposure vs low-exposure states (adjusted β coefficient, −1.6; 95% CI, −2.9 to −0.4), an 80% decline relative to the preperiod mean. This rate was low throughout the study period in low-exposure states, while the rate declined from about 2 per 1000 Medicaid-paid deliveries to less than 0.5 per 1000 Medicaid-paid deliveries in high-exposure states (eFigure 9 in Supplement 1). The rate of hospitalizations paid by private insurance also declined (adjusted β coefficient, −0.2; 95% CI, −0.3 to 0.0), a decrease of 22% from the preperiod mean (0.9; 95% CI, 0.8 to 1.0) in high-exposure states. We did not find significant changes in hospitalization rates with Medicaid or other insurance as the payer.

**Table 3.** Difference-in-Differences Estimates for Hospitalization Rate per 1000 Deliveries 61 to 180 Days Post Partum by Payer Type and Condition^a^

	Estimate (95% CI)			
	States with an uninsured rate at or below the median, preperiod mean	States with an above median uninsured rate, preperiod mean	Unadjusted β coefficient^b^	Adjusted β coefficient^b^^,^^c^
**Expected postpartum hospitalization payer**				
Medicaid	9.2 (9.0 to 9.4)	9.9 (9.6 to 10.2)	0.5 (−0.3 to 1.3)	0.4 (−0.5 to 1.2)
Private	0.4 (0.4 to 0.5)	0.9 (0.8 to 1.0)	−0.2 (−0.3 to 0.0)	−0.2 (−0.3 to 0.0)
Self-pay	0.2 (0.2 to 0.3)	2.0 (1.9 to 2.1)	−1.6 (−2.8 to −0.4)	−1.6 (−2.9 to −0.4)
Other^d^	0.1 (0.1 to 0.2)	0.3 (0.3 to 0.4)	−0.1 (−0.2 to 0.0)	−0.1 (−0.2 to 0.0)
**Postpartum hospitalization condition code**				
K: digestive	3.3 (3.2 to 3.4)	3.0 (2.9 to 3.2)	−0.4 (−1.0 to 0.1)	−0.4 (−1.0 to 0.1)
F: mental^e^	1.5 (1.4 to 1.6)	2.9 (2.8 to 3.0)	0.0 (−0.3 to 0.4)	0.0 (−0.4 to 0.4)
A-B: infection	1.2 (1.1 to 1.2)	1.3 (1.2 to 1.4)	0.0 (−0.3 to 0.3)	−0.1 (−0.4 to 0.3)
O: pregnancy	0.6 (0.5 to 0.6)	0.8 (0.7 to 0.9)	−0.2 (−0.4 to 0.0)	−0.2 (−0.4 to 0.0)
Other^f^	3.8 (3.7 to 4.0)	5.5 (5.3 to 5.7)	−0.6 (−1.1 to 0.0)	−0.7 (−1.4 to 0.0)

^a^ Includes all deliveries with an expected payer of Medicaid (N = 2 024 214).

^b^ Coefficients were estimated from a difference-in-differences model comparing changes in high-exposure vs low-exposure states. High exposure was defined as a preperiod postpartum uninsured rate above the median (13.9%).

^c^ Adjusted models controlled for delivery characteristics, patient demographics and comorbidities, and COVID-19 admission rates in the 61 to 180 days postpartum follow-up period.

^d^ Other expected payer included Medicare, Worker’s Compensation, Civilian Health and Medical Program of the Uniform Services, Civilian Health and Medical Program of the Department of Veterans Affairs, Title V, and other government programs.

^e^ F: mental included mental health and behavioral disorders O9931-O9934.

^f^ Other codes included: C, neoplasms; D, blood and blood-forming organs; E, endocrine; G, nervous system; H, eye and ear; I, circulatory; J, respiratory; L, skin; M, musculoskeletal; N, genitourinary; P, perinatal; Q, congenital; R, symptoms; S-T, injury and poisoning; U, special purposes; V-Y, external causes; Z, factors influencing health status.

In analyses stratified by the most common primary diagnoses for hospitalizations, we did not find statistically significant declines in the rate of hospitalization 61 to 180 days post partum for any category (Table 3). Relative to the baseline means, 95% CIs were wide (eg, −1.0 to 0.1 for digestive), with a 10% reduction falling within the 95% CI for all categories.

During the preperiod, mean postpartum hospitalizations per 1000 Medicaid-paid deliveries in high-exposure states were highest among non-Hispanic White patients (14.6), followed by non-Hispanic Black patients (12.7), Hispanic patients (10.8), and patients with other race or ethnicity (8.8) (Table 4). In stratified analyses, the CCR was associated with declines in hospitalizations 61 to 180 days post partum among non-Hispanic White patients (−1.6 [95% CI, −0.4 to −2.8] hospitalizations per 1000 Medicaid-paid deliveries, which was an 11.0% decrease from the preperiod hospitalization rate 61 to 180 days post partum). The estimate for Hispanic patients was similar in size (adjusted β coefficient, −1.7; 95% CI, −4.3 to 0.8), but not statistically significant. We did not find evidence of significant changes in postpartum hospitalization rates among patients from other racial or ethnic groups.

**Table 4.** Difference-in-Differences Estimates for Hospitalization Rate per 1000 Deliveries 61 to 180 Days Post Partum Stratified by Race, Ethnicity, and County Urbanization

	Estimate (95% CI)			
	States with an uninsured rate at or below the median, preperiod mean	States with an above median uninsured rate, preperiod mean	Unadjusted β coefficient,^a^	Adjusted β coefficient^a^^,^^b^
**Total postpartum hospitalizations stratified by patient race and ethnicity**				
Hispanic (n = 550 881)	10.3 (9.8 to 10.7)	10.8 (10.2 to 11.5)	−1.5 (−3.8 to 0.8)	−1.7 (−4.3 to 0.8)
Non-Hispanic Black (n = 490 586	11.5 (10.9 to 12.2)	12.7 (12.2 to 13.3)	0.2 (−0.6 to 1.1)	0.3 (−0.6 to 1.2)
Non-Hispanic White (n = 744 945)	10.0 (9.6 to 10.4)	14.6 (14.1 to 15.1)	−1.5 (−2.7 to −0.3)	−1.6 (−2.8 to −0.4)
Other (n = 200 639)^c^	6.8 (6.2 to 7.3)	8.8 (7.9 to 9.8)	−0.1 (−2.2 to 2.1)	−0.3 (−2.5 to 1.9)
**Total postpartum hospitalizations stratified by metropolitan status^d^**				
Metropolitan (n = 1 760 708)	10.0 (9.7 to 10.2)	13.0 (12.7 to 13.4)	−1.2 (−2.3 to −0.1)	−1.4 (−2.7 to 0.0)
Nonmetropolitan (n = 263 506)	9.4 (8.6 to 10.2)	12.3 (11.6 to 13.0)	−0.7 (−2.0 to 0.7)	−0.6 (−1.9 to 0.7)

^a^ Coefficients were estimated from a difference-in-differences model comparing changes in high-exposure vs low-exposure states. High exposure was defined as the preperiod postpartum uninsured rate above the median (13.9%). Includes all deliveries with an expected payer of Medicaid.

^b^ Adjusted models controlled for delivery characteristics, patient demographics and comorbidities, and COVID-19 admission rates in the 61 to 180 days post partum follow-up period.

^c^ Other race or ethnicity includes American Indian or Alaska Native, Asian, and other races or ethnicities (except Hispanic, Black, and White).

^d^ Counties classified based on 2023 Rural-Urban Continuum Code.

During the preperiod, mean postpartum hospitalization rates per 1000 Medicaid-paid deliveries were similar for patients who lived in nonmetropolitan vs metropolitan counties (12.3 [95% CI, 11.6 to 13.0] vs 13.0 [95% CI, 12.7 to 13.4]) (Table 4). The CCR was associated with a decline in hospitalization 61 to 180 days post partum for patients from metropolitan counties (adjusted β coefficient, −1.4; 95% CI, −2.7 to 0.0). We did not find evidence of significant changes in postpartum hospitalizations among patients from nonmetropolitan counties, but 95% CIs were large relative to the preperiod means, possibly due to small sample size.

## Discussion

In this cohort study with a DID analysis using data from 20 states, we found that states with higher preperiod postpartum uninsured rates experienced larger declines in postpartum hospitalizations after the CCR extended postpartum Medicaid coverage. Reductions were limited to hospitalizations 61 to 180 days after delivery for patients with Medicaid-paid deliveries. These declines were associated with large decreases in self-pay postpartum hospitalizations, which decreased to close to zero during the postperiod. We did not find significant changes in 2 placebo tests (hospitalizations 1 to 60 days post partum for patients with Medicaid-paid deliveries and postpartum hospitalizations for patients with privately insured deliveries).

The primary group expected to benefit from the CCR was mothers who were covered by Medicaid at delivery but would have lost coverage after 60 days post partum because of lower income eligibility thresholds for parental compared with pregnancy-related Medicaid. Although we did not directly observe patient income or Medicaid eligibility, we found that decreases in postpartum hospitalizations were primarily associated with decreases in self-pay postpartum hospitalizations. This suggests that the reduction in postpartum hospitalizations was largely limited to mothers who would have lost insurance coverage in the absence of the CCR, presumably because they would not have been eligible for Medicaid after 60 days post partum. The association of the CCR with fewer overall hospitalizations suggests that a substantial number of self-pay hospitalizations were averted rather than transitioning to Medicaid-paid hospitalizations.

Related studies have found that the CCR increased postpartum Medicaid coverage and decreased uninsurance.^10,11,12,13,14^ Our finding of declines in hospitalization rates could be explained by improved access to outpatient and other preventive care for mothers who would have lost insurance coverage without the CCR. Evidence on how such care was affected by the CCR is limited, with Wang and colleagues^38^ finding an increase in postpartum outpatient care, and Daw and colleagues^13^ finding no evidence of changes in postpartum care. Analyses of other insurance expansions have found that postpartum coverage increases access to care,^4^ including screening and treatment, which can improve health and reduce the risk of adverse events.^1,2^ Understanding how the CCR affected use of preventive and outpatient care remains an important avenue for future research.

Our study was underpowered to explore differences by patient race and ethnicity, type of condition, and metropolitan vs nonmetropolitan counties. We only found evidence of a decline in hospitalization rate among non-Hispanic White patients and patients residing in metropolitan counties. However, patients of other races and ethnicities and patients in nonmetropolitan counties may have been similarly affected. We were unable to reject a 10.9% reduction in hospitalizations (our main estimate) for patients who were Hispanic, non-Hispanic White, other races or ethnicities, and for patients residing in nonmetropolitan counties (ie, a decrease of that size was within the 95% CI for those subgroups). Similarly, we were unable to reject a 10.9% reduction in hospitalization for any diagnosis category.

Our results suggest that the CCR was associated with reduced differences in hospitalization rates among patients covered by Medicaid and patients covered by private insurance. In the preperiod, the hospitalization rate 61 to 180 days post partum for patients with Medicaid-paid deliveries was more than twice as high as the rate for privately insured deliveries (12.9 vs 5.7 per 1000, respectively). In our placebo analysis, we did not find a change in hospitalizations for privately insured deliveries. The reduction in hospitalization rate for patients with Medicaid-paid deliveries by 1.4 per 1000 deliveries corresponds to 19.4% of the gap between patients with Medicaid-paid deliveries and patients with privately insured deliveries.

The CCR ended March 31, 2023. Since then, more than 24 million individuals in the US have been disenrolled from Medicaid.^39^ However, the American Rescue Plan Act of 2021 allowed states to extend Medicaid coverage to 12 months post partum. As of January 2025, 48 states plus Washington, DC, had implemented this extension.^15^ Our results suggest that this policy may be associated with improved postpartum health outcomes for mothers with low income and reduced differences with mothers who are privately insured.

### Limitations

This study had several limitations. It included 20 states, which could limit the generalizability of our findings if the association differed in other states. Nonetheless, at least one state from each Census division was included, and these states accounted for 45.5% of the US population in 2020. It was not possible to identify hospitalizations that occurred in different states than the state of the delivery admission. We did not observe hospitalizations in federal acute-care hospitals, such as the Indian Health Service, Veteran’s Administration, and Department of Defense.

Our estimates reflect the association of extended postpartum Medicaid coverage with postpartum hospitalizations during the COVID-19 pandemic. Although we took steps to control for the potential confounders related to the pandemic on hospitalizations and examined robustness excluding the initial pandemic months, the association of extended postpartum Medicaid coverage with hospitalization rates may differ in a nonpandemic context, when both outpatient and inpatient care are more accessible.

Researchers are often interested in causal associations of a policy, that is, the difference between the observed outcome and an unobserved counterfactual in which the policy was not implemented. We caution that our main empirical strategy did not estimate that association; rather, we measured the incremental change in high-exposure vs low-exposure states.^24,25^ Measuring the causal association of the policy would have required stronger assumptions that ruled out selection bias (eg, New York would have benefitted from the CCR just as much as Florida did if it had the same level of exposure). Another difference in our estimate is that if the CCR policy was associated with a reduction in postpartum hospitalizations in low-exposure states, then the incremental change in high-exposure vs low-exposure states would be less than the causal association in high-exposure states. Thus, our approach may be a conservative estimate of the CCR’s causal association with postpartum hospitalizations in high-exposure states.

## Conclusion

To our knowledge, this study provides the first evidence that extended Medicaid coverage from 2020 through 2021 was associated with reduced postpartum hospitalizations in the US. This finding suggests that policies that extend postpartum insurance coverage could be associated with improved health, and extended Medicaid coverage may be associated with improved postpartum health for mothers with low income.

## Notes

Supplement 1.
eMethods
**eTable 1.** Included States and Data Sources for Postpartum Insurance Coverage **eFigure 1.** Relationship between PRAMS and ACS uninsurance rates **eTable 2.** Regressions Predicting PRAMS Postpartum Uninsurance Rate using ACS Postpartum Uninsurance Rate **eFigure 2.** Change in Postpartum Uninsurance by Level in Pre-period **eTable 3.** Regressions Predicting Change in PRAMS Uninsurance Rate using the 2018-2019 Uninsurance Rate **eTable 4.** Hospitalizations by Category and Time Since Delivery **eTable 5.** Most Common Diagnosis Codes for Hospitalizations 1-60 days after Delivery **eTable 6.** Most Common Diagnosis Codes for Hospitalizations 61-180 days after Delivery **eFigure 3.** Event Study for Hospitalization within 1-60 days after Delivery **eFigure 4.** Event Study for Hospitalization within 61-180 days after Delivery **eFigure 5.** Unadjusted Change in Hospitalization 61-180 Days after Delivery and Pre-Period Uninsured Rate by State **eFigure 6.** Adjusted Change in Hospitalization 61-180 Days after Delivery and Pre-period Uninsured Rate by State **eTable 7.** Difference-in-Differences Estimates using Continuous Postpartum Uninsurance Rate in Pre-Period **eTable 8.** Difference-in-Differences Estimates Excluding Periods for COVID-19 Pandemic Severity **eTable 9.** Estimates using ACS Postpartum Uninsurance Rate **eTable 10.** Estimates using Continuous ACS Postpartum Uninsured Rate **eFigure 7.** Unadjusted Trends in Hospitalization within 1-60 days of Delivery by Uninsurance Rate in Pre-Period, among Deliveries with Expected Payer Medicaid **eFigure 8.** Unadjusted Trends in Hospitalization within 61-180 days of Delivery by Uninsurance Rate in Pre-Period, among Deliveries with Expected Payer Private Insurance **eFigure 9.** Unadjusted Trends in Hospitalization with Expected Payer Self-Pay within 61-180 days of Delivery by Uninsurance Rate in Pre-Period, among Deliveries with Expected Payer Medicaid **eTable 11.** Difference-in-Differences Placebo Estimates, among Deliveries with Expected Payer Private Insurance

Supplement 2.
Data Sharing Statement
