# Supplementary material for: Medicaid Continuous Coverage Requirement and Postpartum Hospitalization
Source: JAMA Health Forum. 2026 Feb 27;7(2):e256872. doi: 10.1001/jamahealthforum.2025.6872 (PMC12949437; doi:10.1001/jamahealthforum.2025.6872)
Supplement: Supplement 1. — eMethods eTable 1. Included States and Data Sources for Postpartum Insurance Coverage eFigure 1. Relationship between PRAMS and ACS uninsurance rates eTable 2. Regressions Predicting PRAMS Postpartum Uninsurance Rate using ACS Postpartum Uninsurance Rate eFigure 2. Change in Postpartum Uninsurance by Level in Pre-period eTable 3. Regressions Predicting Change in PRAMS Uninsurance Rate using the 2018-2019 Uninsurance Rate eTable 4. Hospitalizations by Category and Time Since Delivery eTable 5. Most Common Diagnosis Codes for Hospitalizations 1-60 days after Delivery eTable 6. Most Common Diagnosis Codes for Hospitalizations 61-180 days after Delivery eFigure 3. Event Study for Hospitalization within 1-60 days after Delivery eFigure 4. Event Study for Hospitalization within 61-180 days after Delivery eFigure 5. Unadjusted Change in Hospitalization 61-180 Days after Delivery and Pre-Period Uninsured Rate by State eFigure 6. Adjusted Change in Hospitalization 61-180 Days after Delivery and Pre-period Uninsured Rate by State eTable 7. Difference-in-Differences Estimates using Continuous Postpartum Uninsurance Rate in Pre-Period eTable 8. Difference-in-Differences Estimates Excluding Periods for COVID-19 Pandemic Severity eTable 9. Estimates using ACS Postpartum Uninsurance Rate eTable 10. Estimates using Continuous ACS Postpartum Uninsured Rate eFigure 7. Unadjusted Trends in Hospitalization within 1-60 days of Delivery by Uninsurance Rate in Pre-Period, among Deliveries with Expected Payer Medicaid eFigure 8. Unadjusted Trends in Hospitalization within 61-180 days of Delivery by Uninsurance Rate in Pre-Period, among Deliveries with Expected Payer Private Insurance eFigure 9. Unadjusted Trends in Hospitalization with Expected Payer Self-Pay within 61-180 days of Delivery by Uninsurance Rate in Pre-Period, among Deliveries with Expected Payer Medicaid eTable 11. Difference-in-Differences Placebo Estimates, among Deliveries with Expected Payer Private Insurance [file jamahealthforum-e256872-s001.pdf]

Supplemental Online Content

Meille G, Steenland MW, Eliason EL. Medicaid continuous coverage requirement and postpartum hospitalization. *JAMA Health Forum.* 2026;7(2): e256872
doi:10.1001/jamahealthforum.2025.6872

eMethods
eTable 1. Included States and Data Sources for Postpartum Insurance Coverage
eFigure 1. Relationship between PRAMS and ACS uninsurance rates
eTable 2. Regressions Predicting PRAMS Postpartum Uninsurance Rate using ACS Postpartum Uninsurance Rate
eFigure 2. Change in Postpartum Uninsurance by Level in Pre-period
eTable 3. Regressions Predicting Change in PRAMS Uninsurance Rate using the 2018-2019 Uninsurance Rate
eTable 4. Hospitalizations by Category and Time Since Delivery
eTable 5. Most Common Diagnosis Codes for Hospitalizations 1-60 days after Delivery
eTable 6. Most Common Diagnosis Codes for Hospitalizations 61-180 days after Delivery
eFigure 3. Event Study for Hospitalization within 1-60 days after Delivery
eFigure 4. Event Study for Hospitalization within 61-180 days after Delivery
eFigure 5. Unadjusted Change in Hospitalization 61-180 Days after Delivery and Pre-Period Uninsured Rate by State
eFigure 6. Adjusted Change in Hospitalization 61-180 Days after Delivery and Pre-period Uninsured Rate by State
eTable 7. Difference-in-Differences Estimates using Continuous Postpartum Uninsurance Rate in Pre-Period
eTable 8. Difference-in-Differences Estimates Excluding Periods for COVID-19 Pandemic Severity
eTable 9. Estimates using ACS Postpartum Uninsurance Rate
eTable 10. Estimates using Continuous ACS Postpartum Uninsured Rate
eFigure 7. Unadjusted Trends in Hospitalization within 1-60 days of Delivery by Uninsurance Rate in Pre-Period, among Deliveries with Expected Payer Medicaid
eFigure 8. Unadjusted Trends in Hospitalization within 61-180 days of Delivery by Uninsurance Rate in Pre-Period, among Deliveries with Expected Payer Private Insurance
eFigure 9. Unadjusted Trends in Hospitalization with Expected Payer Self-Pay within 61-180 days of Delivery by Uninsurance Rate in Pre-Period, among Deliveries with Expected Payer Medicaid
eTable 11. Difference-in-Differences Placebo Estimates, among Deliveries with Expected Payer Private Insurance

© 2026 Meille G et al. *JAMA Health Forum*.

This supplemental material has been provided by the authors to give readers additional information about their work.

© 2026 Meille G et al. *JAMA Health Forum*.

eMethods

A1. Included States and Data Sources for Postpartum Insurance Coverage

eTable 1 shows the data source for the postpartum uninsurance rate. For 14 states 2018-2019 data on postpartum uninsurance for people whose deliveries were covered by Medicaid were available from PRAMS. For 6 states, we imputed the postpartum uninsurance rate using a measure of postpartum uninsurance from the 2018-2019 ACS. The ACS measure was the 2018-2019 uninsurance rate among people who had a birth in the past 12 months and reported income less than their state's Medicaid eligibility levels for pregnancy.

eFigure 1 and eTable 2 show that the ACS measure of postpartum uninsurance was highly predictive of the PRAMS measure (using data from all 38 states where both data sources were available in 2018-2019). We imputed the postpartum uninsurance rate for study states with missing PRAMS data using Specification 2 from eTable 2. This specification weighted observations by the inverse of the standard error of the difference in uninsurance rates as reported by the two surveys. This weighting approach takes into account the accuracy of the surveys, which have different numbers of respondents in different states.

eFigure 2 and eTable 3 show that states with higher pre-PHE uninsurance rates experienced larger declines in uninsurance during the post-period (using data from all 30 states where PRAMS data was available in 2018-2021; note that 8 states were available in 2018-2019 but not 2020-2021). Specification 1 of eTable 3 runs a regression of the change in PRAMS uninsured rate on the pre-period PRAMS uninsured rate. Specification 2 runs an instrumental variables regression, where the ACS uninsured rate is used as the instrument. The instrumental variables approach addresses bias from measurement error, as in Specification 1 measurement error for the pre-period uninsurance rate estimate is included in both the dependent and independent variable. Both approaches show that the pre-PHE uninsurance rates were highly predictive of the change during the post-period, with uninsurance rates declining by about 50% in all states.

A2. Difference-in-differences Model

For our difference-in-differences specification, we estimated the following model:

$$Y_{ist} = \beta * AboveMedianExposure_s * Post_t + \gamma * X_{ist} + \alpha_s + \tau_t$$

Y_{ist} represents our outcome of interest, postpartum hospitalization. $AboveMedianExposure_s$ is an indicator for whether the state 2018-2019 postpartum uninsurance rate among mothers with Medicaid-paid deliveries was above the median rate in our sample. $Post_t$ is an indicator for the CCR exposure period, (2020-2021). Postpartum Medicaid coverage for January 2020 deliveries would have extended to March 2020 under the pre-CCR 60-day regime. Thus, the January 2020 birthing cohort was the first to be affected by the CCR policy implemented in March 2020.

$AboveMedianExposure_s * Post_t$ is the main variable of interest, and β measures the change in postpartum hospitalization in states with above-median pre-PHE postpartum uninsurance relative to states with median or below-median pre-PHE postpartum uninsurance. X_{ist} includes control

variables, including state Medicaid expansion status, length of stay (1, 2, 3, 4+ days), age at delivery (18-24, 25-29, 30-34, 35-39, 40+ years), race and ethnicity (Non-Hispanic Asian, Non-Hispanic Black, Hispanic, Non-Hispanic White, Other, Missing), gestational age (<32, 32-36, 37-38, 39-40, 41+ weeks), cesarean delivery, COVID-19 diagnosis at delivery, Hospital Service Area-level COVID-19 admission rate in the follow-up period, the Elixhauser Comorbidity Index Refined for readmission, an indicator for severe maternal morbidity, and the rural-urban continuum code (1, 2, 3, 4+). α_s represents state fixed effects and τ_t represents year fixed effects. We estimated standard errors using wild bootstrapping.

A3. Continuous Difference-in-Differences Estimates

For our continuous difference-in-differences specification, we estimated the following model:

$$Y_{ist} = \beta * Exposure_{st} * Post_t + \gamma * X_{ist} + \alpha_s + \tau_t$$

In this regression $Exposure_{st}$ is the 2018-2019 postpartum uninsurance rate among people with Medicaid-paid deliveries by state. β measures the change in postpartum hospitalization in states with higher pre-PHE postpartum uninsurance relative to states with lower pre-PHE postpartum uninsurance. All other variables are defined similarly to the main difference-in-differences model, presented in eMethods A2.

Similarly to our main results, we find a statistically significant decline in readmission within 61-180 days and no effect on hospitalization within 60 days (eTable 7). To estimate the average decline in hospitalization within 61-180 days, we multiplied the model coefficient (-9.7 hospitalizations per 1000 deliveries) by the average uninsurance rate in the pre-period (16.3%). This yielded an average decline of 1.6 hospitalizations per 1000 deliveries, a 14.2% decline relative to the pre-period mean. This estimate is similar in size to the estimate from our main model.

A4. Robustness Checks

In eTable 8, we present unadjusted and adjusted estimates for postpartum hospitalization within 61-180 days with no periods excluded (our main models) alongside estimates that omitted deliveries that occurred January-June 2020 and September 2019-June 2020. We excluded deliveries occurring in these windows as the postpartum period coincided with the initial months of the COVID-19 pandemic, which may have affected rates of postpartum hospitalization. Results were robust to omitting these periods, with estimates similar in size and significance to the main results.

We also estimated our main difference-in-difference model and the continuous model using the 2018-2019 ACS postpartum uninsurance rate as the independent variable. These results are similar to the results using the PRAMS rate, although the coefficients for the categorical model are only statistically significant at the 10% level (eTables 9 and 10). This is not surprising, as the ACS data are noisier for smaller states because they tend to have fewer observations than the PRAMS data. Furthermore, the ACS measure is slightly less appropriate than the PRAMS

© 2026 Meille G et al. *JAMA Health Forum*.

measure because the ACS measures uninsurance for people who delivered any time in the past 12 months and the survey does not ask about the type of coverage at the time of delivery.

Finally, we estimated the main difference-in-differences model among an alternate cohort of patients, those with expected payer of private insurance at delivery. These patients would not have been affected by extended postpartum Medicaid coverage during the CCR. Among patients with an expected payer of private coverage at delivery, we found no evidence of changes in postpartum hospitalization (eTable 11). These results support our interpretation that extended postpartum Medicaid coverage drove the changes for patients with expected payer of Medicaid at delivery, as opposed to changes due to the COVID-19 pandemic, which would have also affected patients with private insurance coverage.

© 2026 Meille G et al. *JAMA Health Forum*.

eTable 1. Included States and Data Sources for Postpartum Insurance Coverage

Included states with PRAMS data (14)	AK, AR, IA, LA, MD, MO, MS, NY, OR, SD, VA, VT, WI, WY
Included states with uninsurance imputed from ACS data (6)	CA, FL, IN, NV, SC, TN
States not included (31)	AL, AZ, CO, CT, DE, DC, GA, HI, ID, IL, KS, KY, ME, MA, MI, MN, MT, NE, NH, NJ, NM, NC, ND, OH, OK, PA, RI, TX, UT, WA, WV

© 2026 Meille G et al. *JAMA Health Forum*.

eFigure 1. Relationship between PRAMS and ACS uninsurance rates

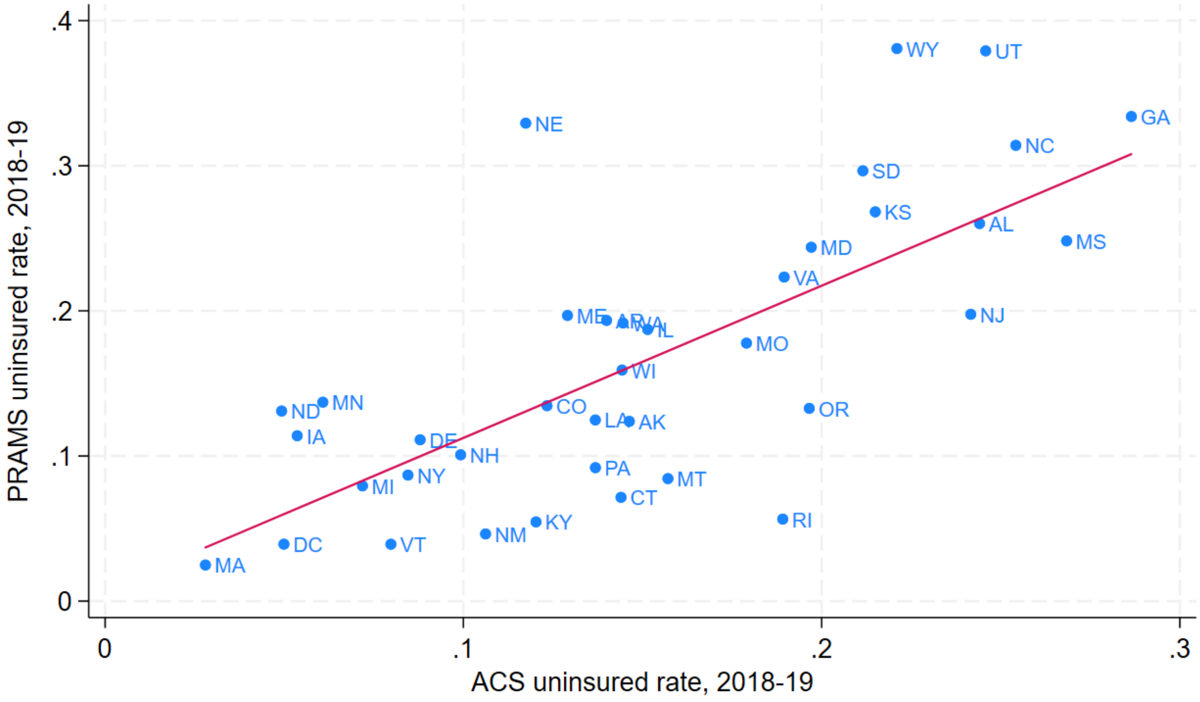

Source: 2018-2019 PRAMS and ACS, 38 states
Includes all states with 2018-2019 PRAMS data; note that 8 states with 2018-2019 data did not have 2020-2021 data. Red line shows the weighted regression (Specification 2) from eTable A2.

© 2026 Meille G et al. *JAMA Health Forum*.

eTable 2. Regressions Predicting PRAMS Postpartum Uninsurance Rate using ACS Postpartum Uninsurance Rate

Variables	Estimate (95% CI)	
	Specification 1	Specification 2
ACS uninsured rate	1.08 (0.74, 1.41)	1.05 (0.76, 1.34)
Constant	0.01 (-0.05, 0.06)	0.01 (-0.04, 0.05)
N	38	38
R-squared	0.538	0.604
Weights	No	Yes

Source: 2018-2019 PRAMS and ACS, 38 states

Includes all states with 2018-2019 PRAMS data; note that 8 states with 2018-2019 data did not have 2020-2021 data. For weighted regression, observations were weighted by the inverse of the standard error of the difference in means for each state.

© 2026 Meille G et al. *JAMA Health Forum*.

eFigure 2. Change in Postpartum Uninsurance by Level in Pre-period

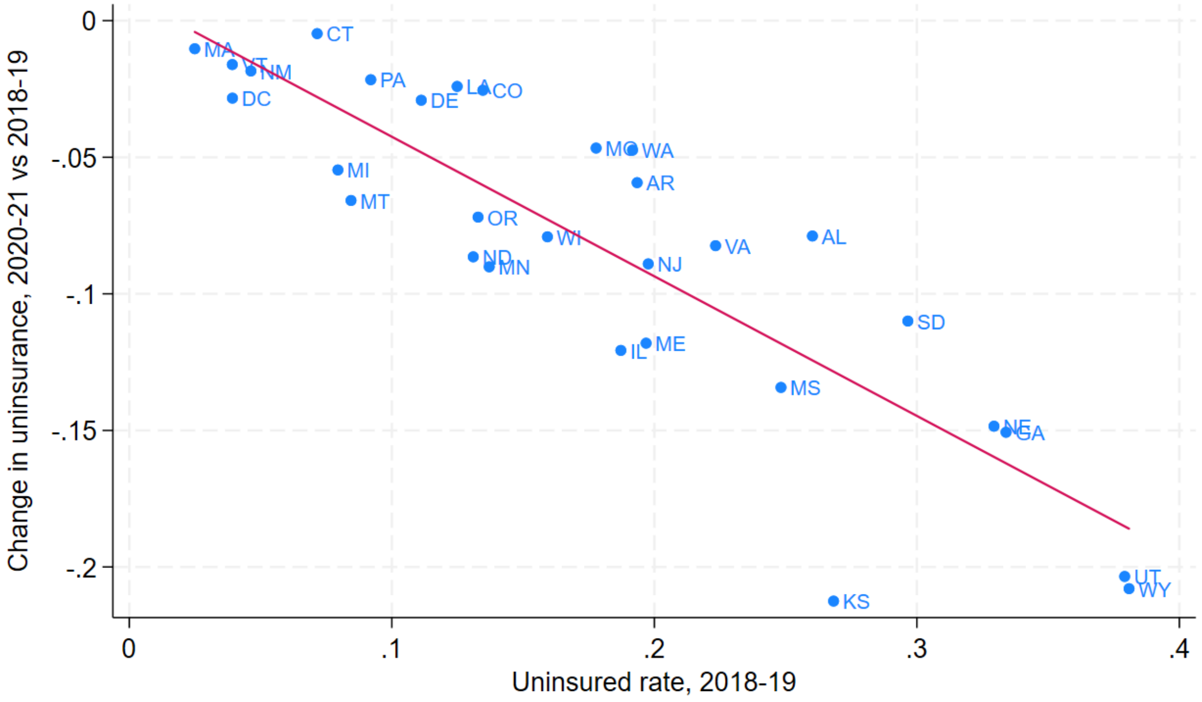

Source: 2018-2021 PRAMS, 30 states
Includes all states with 2018-2021 PRAMS data; note that 8 states with 2018-2019 data did not have 2020-2021 data. Red line shows the results from Specification 1 of eTable A3.

© 2026 Meille G et al. *JAMA Health Forum*.

eTable 3. Regressions Predicting Change in PRAMS Uninsurance Rate using the 2018-2019 Uninsurance Rate

Variables	Estimate (95% CI)	
	Specification 1	Specification 2[a]
PRAMS 2018-2019 uninsured rate	-0.51 (-0.62, -0.40)	-0.47 (-0.62, -0.33)
Constant	0.01 (-0.01, 0.03)	0.00 (-0.02, 0.03)
N	30	30
R-squared	0.757	0.754
Method	Linear regression	2 stage least squares

Source: 2018-2021 PRAMS and 2018-2019 ACS, 30 states

Includes all states with 2018-2021 PRAMS data; note that 8 states with 2018-2019 data did not have 2020-2021 data.

[a]2 stage least squares estimate, using the ACS uninsured rate as an instrumental variable.

© 2026 Meille G et al. *JAMA Health Forum*.

eTable 4. Hospitalizations by Category and Time Since Delivery

Category	1-60 days postpartum		61-180 days postpartum	
	No.	%	No.	%
A-B. Infections and parasitic diseases	1494	1.6	3721	9.3
C00-D49. Neoplasms	334	0.4	1168	2.9
D50-D89. Blood diseases	247	0.3	967	2.4
E. Endocrine, nutritional, and metabolic diseases	313	0.3	1471	3.7
F. Mental and behavioral disorders[a]	4371	4.7	6698	16.8
G. Nervous system diseases	402	0.4	1041	2.6
I. Circulatory diseases	1092	1.2	2009	5.0
J. Respiratory diseases	532	0.6	1217	3.1
K. Digestive diseases	5503	5.9	11114	27.9
L. Skin diseases	321	0.3	667	1.7
M. Musculoskeletal diseases	172	0.2	633	1.6
N. Genitourinary diseases	1389	1.5	2703	6.8
O. Pregnancy, childbirth, and puerperium	74911	79.7	2024	5.1
R. Symptoms, signs, and clinical and lab findings	400	0.4	849	2.1
S-T. Injury and poisoning	1124	1.2	2725	6.8
Other (H, P, Q, U, Z)	1392	1.5	886	2.2

Source: AHRQ Healthcare Cost and Utilization Project, 2018-2021 State Inpatient Databases, 20 states

[a]Mental health and behavioral disorders O9931-O9934 included in F.

© 2026 Meille G et al. *JAMA Health Forum*.

eTable 5. Most Common Diagnosis Codes for Hospitalizations 1-60 days after Delivery

Code	Condition	Count	Percentage
O1415	Severe pre-eclampsia, complicating the puerperium	15603	16.6%
O1495	Unspecified pre-eclampsia, complicating the puerperium	7761	8.3%
O8612	Endometritis following delivery	5445	5.8%
O9963	Diseases of the digestive system complicating the puerperium	4295	4.6%
O115	Pre-existing hypertension with pre-eclampsia, complicating the puerperium	3952	4.2%
O85	Puerperal sepsis	3353	3.6%
O9089	Other complications of the puerperium, not elsewhere classified	2253	2.4%
O722	Delayed and secondary postpartum hemorrhage	2166	2.3%
O8601	Infection of obstetric surgical wound, superficial incisional site	1730	1.8%
O99345	Other mental disorders complicating the puerperium	1570	1.7%
other	Other	45869	48.8%

Source: AHRQ Healthcare Cost and Utilization Project, 2018-2021 State Inpatient Databases, 20 states

© 2026 Meille G et al. *JAMA Health Forum*.

eTable 6. Most Common Diagnosis Codes for Hospitalizations 61-180 days after Delivery

Code	Condition	Count	Percentage
A419	Sepsis, unspecified organism	2324	5.8%
K8000	Calculus of gallbladder with acute cholecystitis w/o obstruction	1474	3.7%
K8510	Biliary acute pancreatitis w/o necrosis or infection	1126	2.8%
F332	Major depressive disorder, recurrent severe w/o psychotic features	907	2.3%
E1010	Type 1 diabetes mellitus with ketoacidosis w/o coma	595	1.5%
D5700	Hb-SS disease with crisis, unspecified	586	1.5%
K8062	Calculus of gallbladder and bile duct with acute cholecystitis w/o obstruction	504	1.3%
K3580	Unspecified acute appendicitis	443	1.1%
F329	Major depressive disorder, single episode, unspecified	433	1.1%
K8020	Calculus of gallbladder w/o cholecystitis w/o obstruction	433	1.1%
other	Other	31068	77.9%

Abbreviation: w/o, without
Source: AHRQ Healthcare Cost and Utilization Project, 2018-2021 State Inpatient Databases, 20 states

© 2026 Meille G et al. *JAMA Health Forum*.

eFigure 3. Event Study for Hospitalization within 1-60 days after Delivery

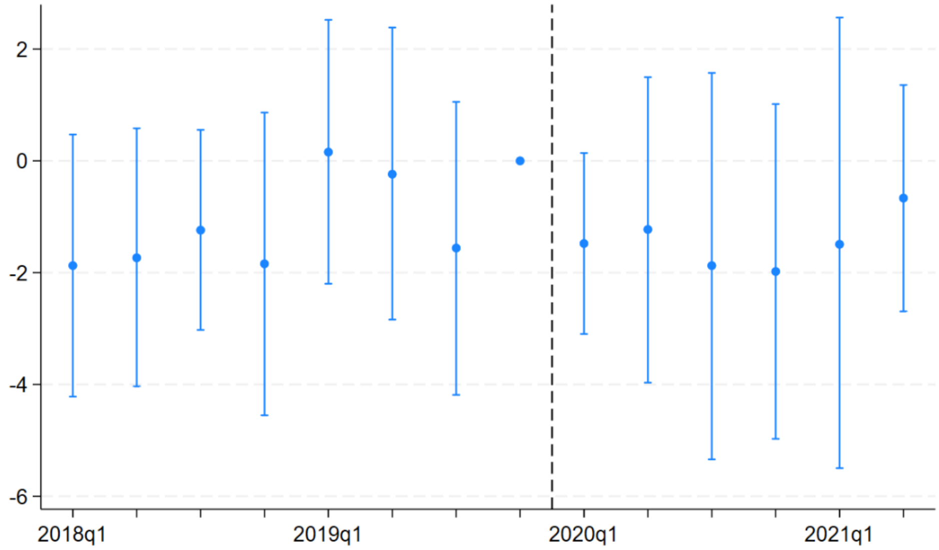

Source: AHRQ Healthcare Cost and Utilization Project, 2018-2021 State Inpatient Databases, 20 states

Notes: Differences in postpartum hospitalization between states with pre-PHE uninsurance rates above or below the median of 13.9%. Deliveries from the last quarter of 2019, when postpartum coverage would have been unaffected by the CCR, are normalized to 0.

© 2026 Meille G et al. *JAMA Health Forum*.

eFigure 4. Event Study for Hospitalization within 61-180 days after Delivery

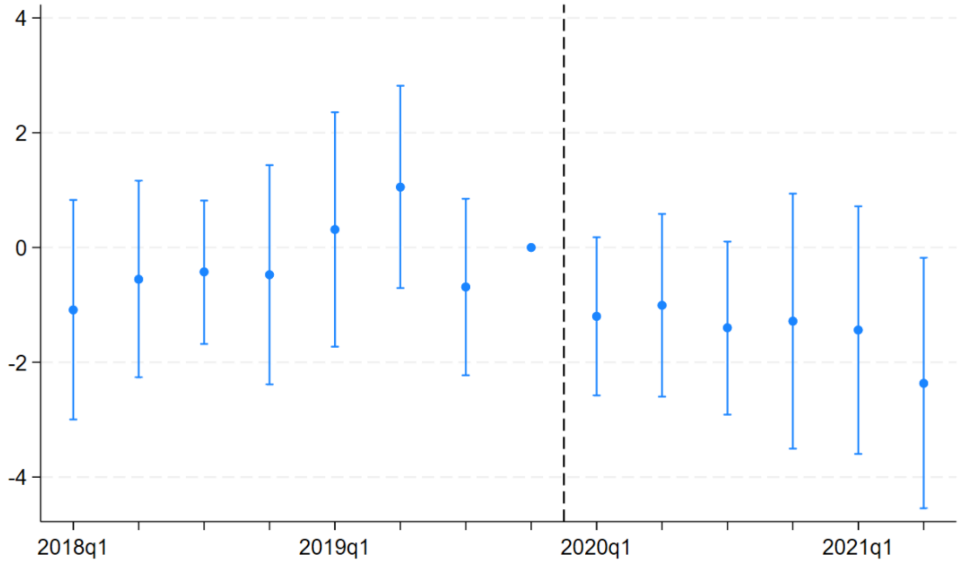

Source: AHRQ Healthcare Cost and Utilization Project, 2018-2021 State Inpatient Databases, 20 states

Notes: Differences in postpartum hospitalization between states with pre-PHE uninsurance rates above or below the median of 13.9%. Deliveries from the last quarter of 2019, when postpartum coverage would have been unaffected by the CCR, are normalized to 0.

© 2026 Meille G et al. *JAMA Health Forum*.

eFigure 5. Unadjusted Change in Hospitalization 61-180 Days after Delivery and Pre-Period Uninsured Rate by State

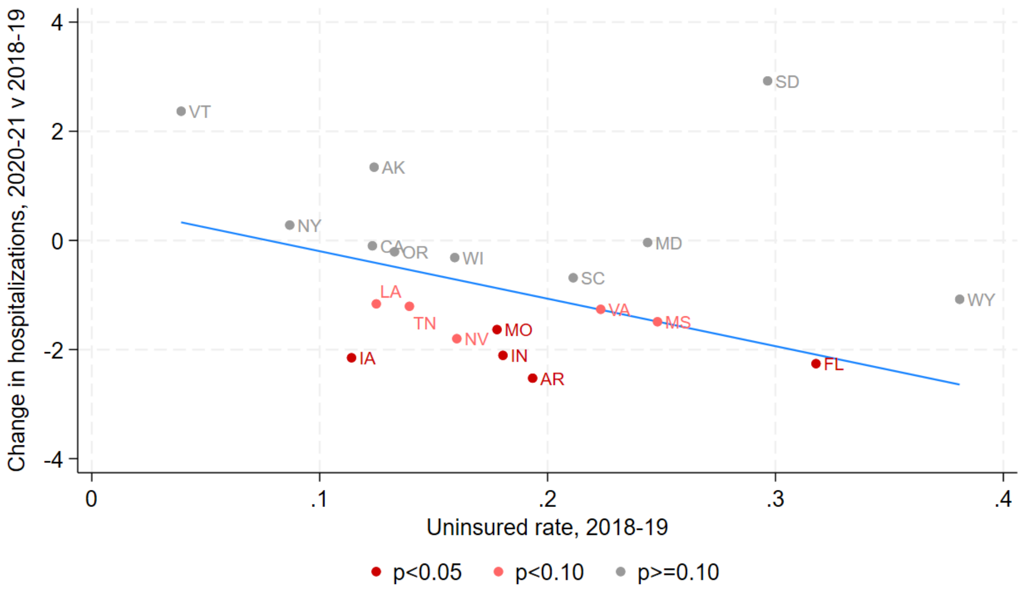

Source: AHRQ Healthcare Cost and Utilization Project, 2018-2021 State Inpatient Databases, 20 states

Line corresponds to regression of change in hospitalization on uninsured rate weighted by number of admissions in each state. Adjusted models controlled for delivery characteristics, patient demographics and comorbidities, and COVID-19 admission rates in the follow-up period.

© 2026 Meille G et al. *JAMA Health Forum*.

eFigure 6. Adjusted Change in Hospitalization 61-180 Days after Delivery and Pre-period Uninsured Rate by State

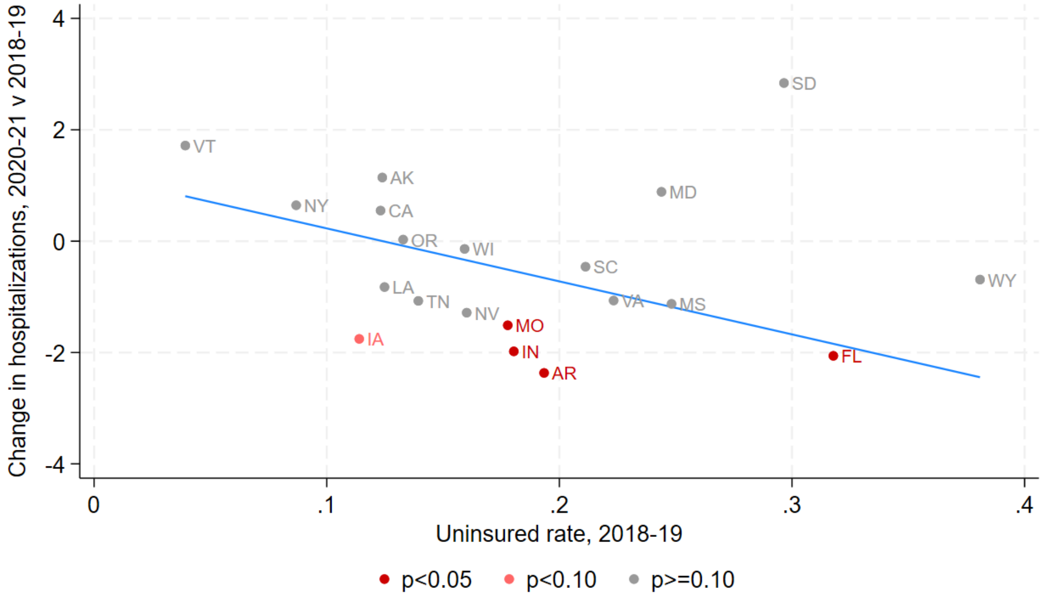

Source: AHRQ Healthcare Cost and Utilization Project, 2018-2021 State Inpatient Databases, 20 states
Line corresponds to regression of change in hospitalization on uninsured rate weighted by number of admissions in each state. Adjusted models controlled for delivery characteristics, patient demographics and comorbidities, and COVID-19 admission rates in the follow-up period.

© 2026 Meille G et al. *JAMA Health Forum*.

eTable 7. Difference-in-Differences Estimates using Continuous Postpartum Uninsurance Rate in Pre-Period

Outcome	Estimate (95% CI)		
	Baseline mean	Coefficient, unadjusted	Coefficient, adjusted
Hospitalization 1-60 days postpartum	20.7<br>(20.4, 20.9)	-1.8<br>(-15.3, 11.8)	-3.5<br>(-18.8, 11.8)
Hospitalization 61-180 days postpartum	11.3<br>(11.1, 11.5)	-8.6<br>(-14.4, -2.8)	-9.6<br>(-17.0, -2.1)

Source: AHRQ Healthcare Cost and Utilization Project, 2018-2021 State Inpatient Databases, 20 states

Exposure variable is 2018-2019 postpartum uninsured rate among deliveries covered by Medicaid. Postpartum hospitalization rate measured per 1000 deliveries. Includes all deliveries with an expected payer of Medicaid. Adjusted models controlled for delivery characteristics, patient demographics and comorbidities, and COVID-19 admission rates in the follow-up period.
N = 2,024,214

© 2026 Meille G et al. *JAMA Health Forum*.

eTable 8. Difference-in-Differences Estimates Excluding Periods for COVID-19 Pandemic Severity

	(1)	(2)	(3)	(4)	(5)	(6)
Coefficient	-1.2	-1.4	-1.4	-1.4	-1.5	-1.6
95% CI	(-2.1, -0.3)	(-2.4, -0.4)	(-2.4, -0.4)	(-2.5, -0.3)	(-2.8, -0.3)	(-2.9, -0.3)
N	2,024,214	1,747,490	1,550,427	2,024,214	1,747,490	1,550,427
Adjusted	No	No	No	Yes	Yes	Yes
Exclusions	None	2020m1-m6	2019m9-2020m6	None	2020m1-m6	2019m9-2020m6

Source: AHRQ Healthcare Cost and Utilization Project, 2018-2021 State Inpatient Databases, 20 states
Coefficients were estimated from a difference-in-differences model comparing changes in high vs. low-exposure states. States were considered highly exposed if the pre-period postpartum uninsurance rate was above the median (13.9 percent). Readmission rate measured per 1000 deliveries. Includes all deliveries with an expected payer of Medicaid. Adjusted models controlled for delivery characteristics, patient demographics and comorbidities, and COVID-19 admission rates in the follow-up period.

© 2026 Meille G et al. *JAMA Health Forum*.

eTable 9. Estimates using Categorical ACS Postpartum Uninsured Rate

Outcome	Estimate (95% CI)			
	Baseline mean, States with uninsured rate at or below the median	Baseline mean, States with above median uninsured rate	Coefficient, unadjusted	Coefficient, adjusted
Hospitalization 1-60 days postpartum	19.8 (19.4, 20.1)	21.6 (21.2, 22.0)	-0.3 (-1.3, 0.7)	-0.6 (-1.8, 0.5)
Hospitalization 61-180 days postpartum	10.1 (9.9, 10.4)	12.6 (12.3, 12.8)	-0.9 (-2.0, 0.1)	-1.1 (-2.3, 0.1)

Source: AHRQ Healthcare Cost and Utilization Project, 2018-2021 State Inpatient Databases, 20 states

Postpartum hospitalization rates measured per 1000 deliveries. Includes all deliveries with an expected payer of Medicaid. Adjusted models controlled for delivery characteristics, patient demographics and comorbidities, and COVID-19 admission rates in the follow-up period.

N = 2,024,214

eTable 10. Estimates using Continuous ACS Postpartum Uninsured Rate

Outcome	Estimate (95% CI)		
	Baseline mean	Coefficient, unadjusted	Coefficient, adjusted
Hospitalization 1-60 days postpartum	20.7 (20.4, 20.9)	-1.4 (-17.1, 14.4)	-3.4 (-20.9, 14.2)
Hospitalization 61-180 days postpartum	11.3 (11.1, 11.5)	-9.0 (-15.8, -2.2)	-10.2 (-18.2, -2.2)

Source: AHRQ Healthcare Cost and Utilization Project, 2018-2021 State Inpatient Databases, 20 states
Postpartum hospitalization rates measured per 1000 deliveries. Includes all deliveries with an expected payer of Medicaid. Adjusted models controlled for delivery characteristics, patient demographics and comorbidities, and COVID-19 admission rates in the follow-up period.
N = 2,024,214

© 2026 Meille G et al. *JAMA Health Forum*.

eFigure 7. Unadjusted Trends in Hospitalization within 1-60 days of Delivery by Uninsurance Rate in Pre-Period, among Deliveries with Expected Payer Medicaid

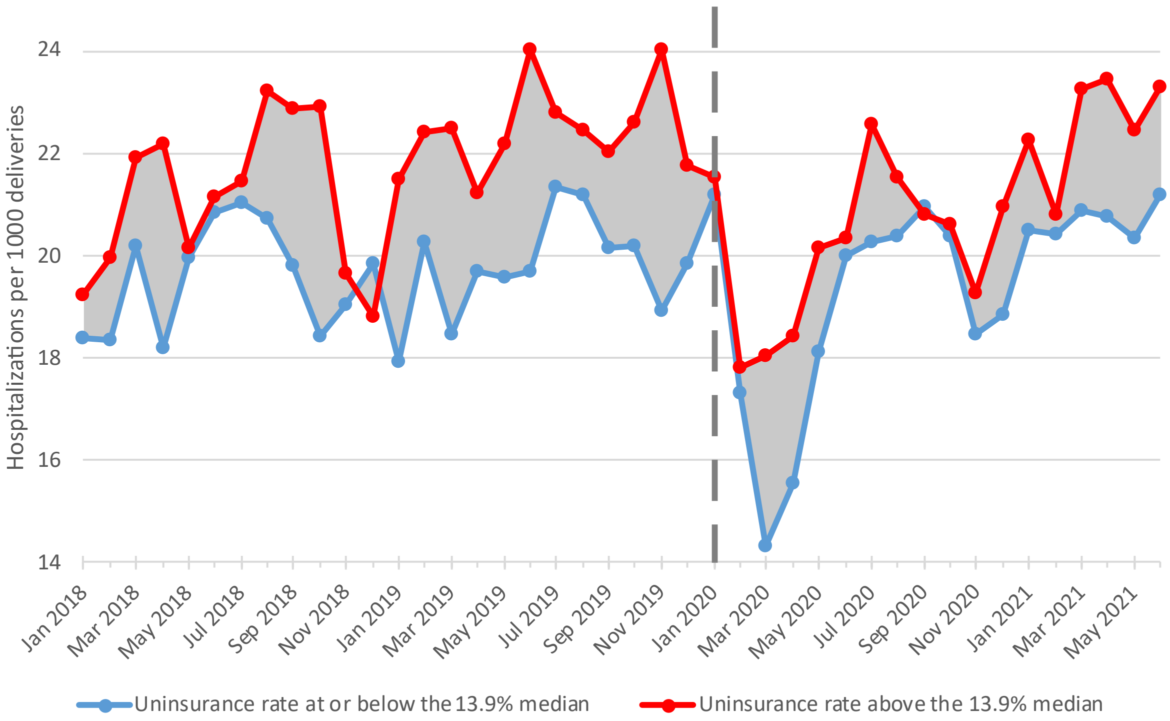

Source: Healthcare Cost and Utilization Project, 2018-2021 State Inpatient Databases, 20 states
Dashed vertical line represents the beginning of the post-period in January 2020. Deliveries during the post-period were affected by the Medicaid continuous coverage requirement.
States with uninsured rate of 0-14%: AK, CA, IA, LA, NY, OR, TN, VT
States with uninsured rate of 14%+: AR, FL, IN, MD, MO, MS, NV, SC, SD, VA, WI, WY

© 2026 Meille G et al. *JAMA Health Forum*.

eFigure 8. Unadjusted Trends in Hospitalization within 61-180 days of Delivery by Uninsurance Rate in Pre-Period, among Deliveries with Expected Payer Private Insurance

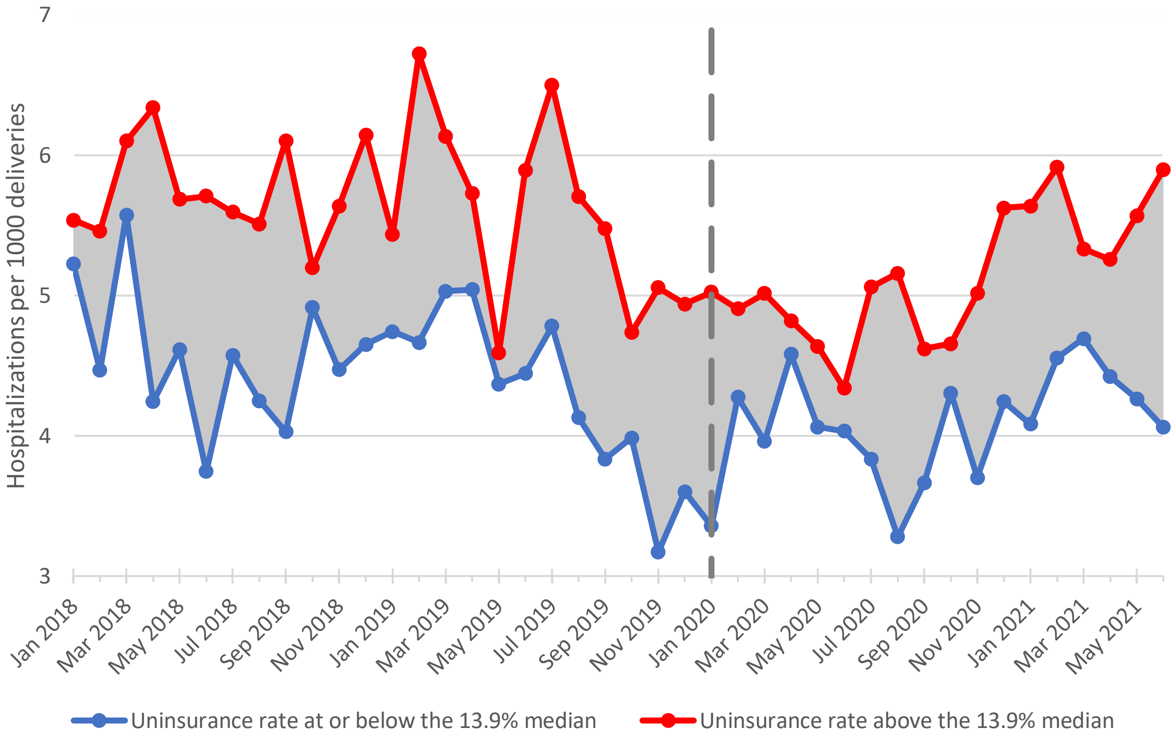

Source: Healthcare Cost and Utilization Project, 2018-2021 State Inpatient Databases, 20 states
Dashed vertical line represents the beginning of the post-period in January 2020. Deliveries during the post-period were affected by the Medicaid continuous coverage requirement.
States with uninsured rate of 0-14%: AK, CA, IA, LA, NY, OR, TN, VT
States with uninsured rate of 14%+: AR, FL, IN, MD, MO, MS, NV, SC, SD, VA, WI, WY

© 2026 Meille G et al. *JAMA Health Forum*.

eFigure 9. Unadjusted Trends in Hospitalization with Expected Payer Self-Pay within 61-180 days of Delivery by Uninsurance Rate in Pre-Period, among Deliveries with Expected Payer Medicaid

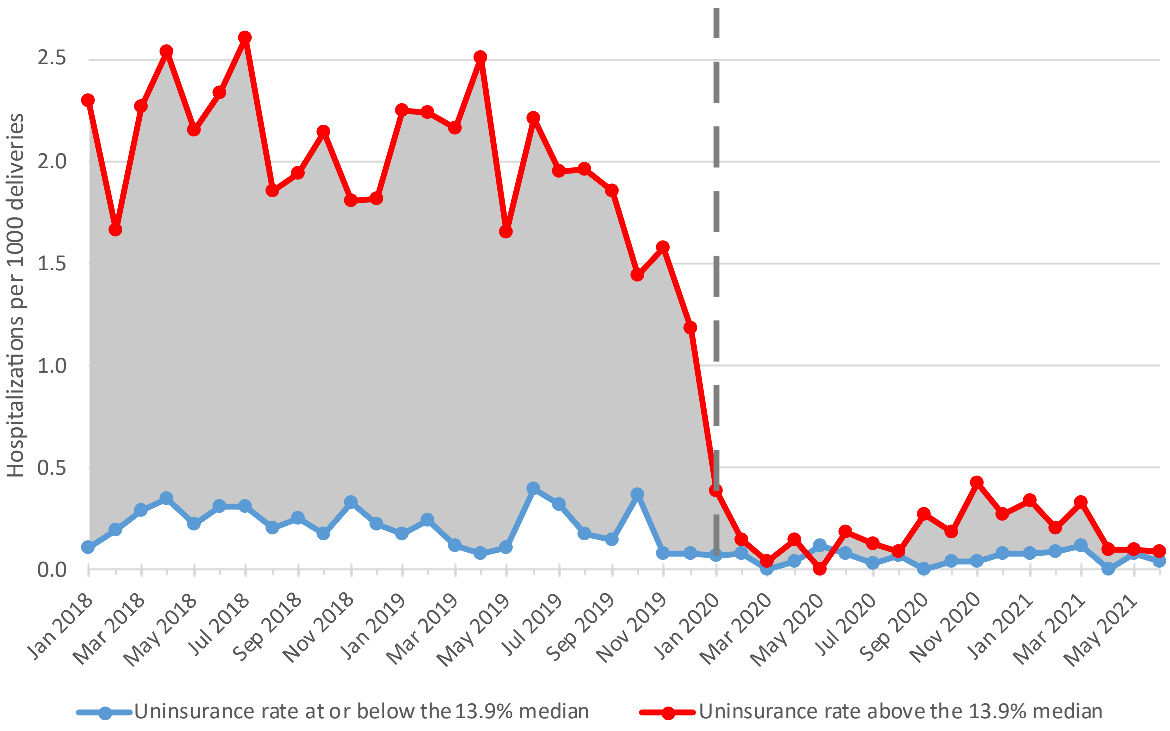

Source: Healthcare Cost and Utilization Project, 2018-2021 State Inpatient Databases, 20 states
Dashed vertical line represents the beginning of the post-period in January 2020. Deliveries during the post-period were affected by the Medicaid continuous coverage requirement.
States with uninsured rate of 0-14%: AK, CA, IA, LA, NY, OR, TN, VT
States with uninsured rate of 14%+: AR, FL, IN, MD, MO, MS, NV, SC, SD, VA, WI, WY

© 2026 Meille G et al. *JAMA Health Forum*.

eTable 11. Difference-in-Differences Placebo Estimates, among Deliveries with Expected Payer Private Insurance

Outcome	Estimate (95% CI)			
	Baseline mean, States with uninsured rate at or below the median	Baseline mean, States with above median uninsured rate	Coefficient, unadjusted	Coefficient, adjusted
Hospitalization 1-60 days postpartum	16.3 (16.0, 16.6)	15.9 (15.6, 16.1)	-0.2 (-1.0, 0.7)	-0.5 (-1.4, 0.5)
Hospitalization 61-180 days postpartum	4.4 (4.3, 4.6)	5.7 (5.5, 5.8)	-0.2 (-0.6, 0.3)	-0.2 (-0.6, 0.2)

Source: AHRQ Healthcare Cost and Utilization Project, 2018-2021 State Inpatient Databases, 20 states
Coefficients were estimated from a difference-in-differences model comparing changes in high vs. low-exposure states. States were considered highly exposed if the pre-period postpartum uninsurance rate was above the median (13.9 percent). Readmission rate measured per 1000 deliveries. Includes all deliveries with an expected payer of private insurance.
N = 2,570,692

© 2026 Meille G et al. *JAMA Health Forum*.
