# Supplementary material for: Medicaid Continuous Coverage Requirement and Postpartum Hospitalization
Source: JAMA Health Forum. 2026 Feb 27;7(2):e256872. doi: 10.1001/jamahealthforum.2025.6872 (PMC12949437; doi:10.1001/jamahealthforum.2025.6872)
Supplement: Supplement 2. — Data Sharing Statement [file jamahealthforum-e256872-s002.pdf]

Data Sharing Statement

Meille. Medicaid Continuous Coverage Requirement and Postpartum Hospitalization. *JAMA Health Forum*. Published February 27, 2026. doi:10.1001/jamahealthforum.2025.6872

Data
Data available: No

Additional Information
Explanation for why data not available: This study uses restricted data from the Healthcare Cost and Utilization Project's (HCUP) State Inpatient Databases (SID). All analyses were completed by Giacomo Meille while he was employed at the Agency for Healthcare Research and Quality. Data for some of the included states is publicly available for purchase from HCUP.
